# Treating the Patient, Not Only the Amyloid: Symptomatic Management in Transthyretin Amyloidosis

**DOI:** 10.3390/neurolint18030053

**Published:** 2026-03-13

**Authors:** Christian Messina

**Affiliations:** Azienda Sanitaria Provinciale Catania, Via Santa Maria La Grande, 95100 Catania, Italy; chry.messina@gmail.com

**Keywords:** ATTR, symptomatologic management, quality of life, autonomic dysfunction

## Abstract

Transthyretin amyloidosis (ATTR) is a progressive multisystem disorder characterized by extracellular deposition of misfolded transthyretin fibrils, leading to neurological, cardiac, gastrointestinal, urogenital, sexual, and ophthalmological involvement. While disease-modifying therapies have significantly improved survival and slowed disease progression, a substantial proportion of patients continue to experience a high symptomatic burden that markedly impairs quality of life. Symptomatic manifestations often occur early, may precede the diagnosis, and frequently persist despite etiological treatment. This review provides a comprehensive overview of the symptomatic management of ATTR, with particular emphasis on autonomic dysfunction and its systemic consequences. We discuss current therapeutic strategies for orthostatic hypotension, gastrointestinal dysmotility, nutritional impairment, sexual dysfunction, lower urinary tract dysfunction, and ophthalmological involvement, highlighting both pharmacological and non-pharmacological approaches. Special attention is given to treatment limitations related to cardiac involvement, autonomic failure, and drug tolerability. Despite the clinical relevance of symptom control in ATTR, evidence-based recommendations remain scarce, and no dedicated guidelines currently exist. Most therapeutic approaches are derived from observational studies, expert opinion, and clinical experience. Improved awareness of symptomatic manifestations, early intervention, and a multidisciplinary, individualized approach are essential to optimize patient outcomes. Future research should focus on prospective studies and the development of structured symptomatic treatment algorithms to complement disease-modifying therapies and enhance patient-centered care in ATTR.

## 1. Introduction

Transthyretin (TTR) amyloidosis (ATTR) is a rare, progressive multisystem disorder caused by the extracellular deposition of amyloid fibrils derived from misfolded TTR protein in various organs and tissues [1,2]. According to the nomenclature recommendations of the International Society of Amyloidosis, ATTR amyloidosis is classified into two main forms: wild-type ATTR (ATTRwt) amyloidosis and hereditary (variant) ATTR (ATTRv) amyloidosis, the latter resulting from pathogenic mutations in the *TTR* gene [3]. ATTR arises from the destabilization of the native TTR tetramer, resulting in its dissociation into individual monomeric subunits, a process widely regarded as the rate-limiting step in disease pathogenesis [4]. Once dissociated, TTR monomers are structurally unstable and prone to misfolding [4]. These misfolded monomers undergo pathological conformational rearrangements that expose normally buried hydrophobic domains, facilitating aberrant intermolecular interactions [4]. Such structural changes favor the formation of β-sheet-rich assemblies and promote the generation of soluble oligomeric species, which are thought to play a key role in early tissue toxicity [4]. Over time, these oligomers progressively aggregate into insoluble amyloid fibrils that accumulate extracellularly within affected tissues [4]. Amyloid deposition most commonly involves peripheral nerves, the myocardium, the gastrointestinal tract, kidneys, and ocular structures, reflecting the multisystem nature of the disease [4]. The progressive accumulation of amyloid fibrils disrupts normal tissue architecture, impairs cellular function, and alters microvascular and extracellular matrix homeostasis [4]. These pathological changes ultimately lead to organ dysfunction and drive the diverse clinical manifestations observed in patients with ATTR [4,5,6]. The disease exhibits marked phenotypic heterogeneity, which significantly influences both clinical presentation and disease trajectory [1,2,4,5,6]. Given its multisystem involvement and clinical complexity, the management of ATTR amyloidosis requires a structured multidisciplinary approach, involving cardiologists, neurologists, internists, and other specialists according to organ involvement [7]. Such coordination is particularly crucial not only for disease-modifying strategies but also for the comprehensive symptomatic management of affected patients. Clinically, ATTR is commonly classified into three major phenotypes: a predominantly cardiologic form, a predominantly neurologic form, and a mixed phenotype with overlapping cardiac and neurologic involvement [1,2]. The diagnosis of ATTR remains challenging, largely due to the nonspecific nature of its symptoms and their frequent overlap with more prevalent cardiovascular and neurological conditions, often leading to diagnostic delay [1,2]. In the absence of timely diagnosis and appropriate therapeutic intervention, ATTR is associated with a poor prognosis, with an estimated life expectancy ranging from approximately 3 to 15 years from symptom onset [2]. Consequently, early recognition, accurate diagnosis, and prompt initiation of treatment are crucial to improve both survival and quality of life [1,2]. Despite substantial advances in disease-modifying therapies targeting TTR production, stabilization, or amyloid deposition, the optimal clinical management of patients with ATTRv, particularly those presenting with polyneuropathy (PN) and multisystem involvement, remains insufficiently standardized [1,2,4]. Currently, evidence-based guidelines specifically addressing the symptomatic treatment of avoiding disease-related manifestations are lacking, resulting in significant variability in clinical practice. This comprehensive review aims to provide clinicians with a practical, symptom-oriented framework for the management of patients with ATTR. By systematically addressing the most common and disabling disease-related symptoms, this review seeks to guide the selection of the most appropriate pharmacological and supportive interventions, thereby promoting a more personalized, rational, and effective approach to symptomatic care in this complex and heterogeneous condition.

## 2. Materials and Methods

This manuscript was conceived as a comprehensive review based on a structured literature search. A systematic search of the PubMed/MEDLINE database was performed to identify relevant publications addressing transthyretin (ATTR) amyloidosis and its symptomatic management. Articles published between January 2000 and March 2026 were considered. The search strategy included combinations of the following keywords: “transthyretin amyloidosis”, “ATTR”, “ATTRv”, “ATTRwt”, “symptomatic treatment”, “autonomic dysfunction”, “polyneuropathy”, “cardiomyopathy”, “gastrointestinal involvement”, “sexual dysfunction”, “ocular involvement”, and “multidisciplinary management”. The following types of publications were included: review articles, clinical trials, observational studies, original research articles, consensus statements, case series, and case reports. Only articles published in English were considered. Evidence selection was guided by relevance to symptomatic management, methodological quality, and clinical applicability, in accordance with the patient-centered focus of this review.

## 3. Pain

Pain represents the most prevalent and clinically impactful symptom in ATTR-related polyneuropathy (ATTR-PN), substantially contributing to impaired health-related quality of life [8]. Approximately 70% of patients with ATTRv report significant pain or pain-related discomfort, underscoring its central role in the overall disease burden [8]. The pain phenotype is typically neuropathic in nature and encompasses a wide spectrum of sensory disturbances, including electric shock-like sensations, tingling, pins-and-needles, burning pain, and pruritus [8]. Neuropathic pain is particularly prominent in patients with early-onset ATTRv, in whom small-fiber involvement is often an early pathological feature [8]. However, clinically relevant pain is also frequently observed in late-onset disease, affecting up to approximately 70% of patients, and has been reported, albeit less commonly, even among asymptomatic or minimally symptomatic mutation carriers, with an estimated prevalence of around 8% [8]. These observations highlight that pain may emerge across different disease stages and phenotypic presentations. Importantly, worsening pain severity appears to correlate with the progression of peripheral neuropathy, suggesting that pain not only reflects symptom burden but may also serve as a clinical marker of disease evolution [8]. As a consequence, pain is one of the most frequently reported and treated symptoms in patients with ATTR, representing a primary therapeutic target in symptomatic management strategies aimed at improving daily functioning and quality of life. According to the most recent clinical guidelines, the management of neuropathic pain relies on a stepwise pharmacological approach that includes first- and second-line agents. Among first-line treatments, gabapentinoids are the most widely prescribed drugs [1,9]. Pregabalin, typically administered at daily doses ranging from 300 to 600 mg, and gabapentin, used at doses between 1200 and 3600 mg per day, are structurally related compounds with comparable mechanisms of action [1,9]. Although commonly referred to as γ-aminobutyric acid (GABA) analogues, gabapentinoids do not exert their analgesic effects through direct interaction with GABA receptors. Instead, they selectively bind to the α2δ subunit of voltage-gated calcium channels, leading to a reduction in calcium influx at presynaptic terminals and a subsequent decrease in neuronal excitability and neurotransmitter release [1,9]. Owing to their established efficacy and extensive use across a broad range of neuropathic pain conditions, gabapentinoids are generally considered well tolerated [1,9]. Nevertheless, their use may be associated with adverse effects such as sedation, dizziness, and peripheral oedema [1,9]. These side effects are of particular clinical relevance in patients with ATTR, who often present with gait instability, balance impairment, and autonomic dysfunction, including orthostatic hypotension [10,11]. Therefore, gabapentinoids should be prescribed with caution in this population, with careful dose titration and close monitoring to minimize the risk of falls and functional deterioration. Other first-line pharmacological options for the treatment of neuropathic pain include tricyclic antidepressants (TCAs), such as amitriptyline and nortriptyline, which are typically prescribed at daily doses ranging from 25 to 150 mg [1,9]. TCAs represent an effective alternative to gabapentinoids and have long been used in the management of neuropathic pain due to their modulation of descending inhibitory pain pathways [1,9]. However, their clinical use is limited by a well-recognized anticholinergic burden (constipation, orthostatic hypotension) and potential cardiotoxic effects [1,9,12]. TCAs are contraindicated in patients with a recent history of myocardial infarction, atrioventricular conduction disturbances, clinically significant arrhythmias, or established coronary artery disease [1,9]. Consequently, these agents should be prescribed with particular caution in patients with a predominantly cardiologic or mixed ATTR phenotype, as they may exacerbate underlying cardiac dysfunction and increase the risk of adverse cardiovascular events. Serotonin–noradrenaline reuptake inhibitors (SNRIs), including venlafaxine and duloxetine, also represent first-line therapeutic options for neuropathic pain [1,9]. Compared with TCAs, SNRIs generally exhibit a more favorable safety and tolerability profile, making them particularly suitable for patients with coexisting mood disorders such as anxiety or depression, which are common in chronic multisystem diseases [1,9]. Nonetheless, potential adverse effects should be carefully considered; in particular, duloxetine may be associated with elevations in blood pressure and, in susceptible individuals, may precipitate uncontrolled hypertensive episodes [1,9]. Therefore, careful patient selection and regular monitoring are essential when SNRIs are used in the ATTR population. Among second-line pharmacological options, sodium channel-blocking antiepileptic drugs such as carbamazepine and oxcarbazepine may be considered for the management of neuropathic pain [1,9,12]. These agents, which are widely used in other neurological conditions, exert their analgesic effect by inhibiting voltage-gated sodium channels, thereby reducing ectopic neuronal firing and hyperexcitability [1,9,12]. They may be particularly effective in neuropathic pain with prominent irritative or paroxysmal features [13]. Despite their potential efficacy, the safety profile of carbamazepine and oxcarbazepine is less favorable, especially in patients with ATTR. Carbamazepine is contraindicated in individuals with atrioventricular conduction abnormalities, a clinically relevant limitation in a population frequently affected by cardiac involvement. Moreover, both agents are associated with a range of adverse effects, including nausea, vomiting, abdominal discomfort, gait disturbances, and hyponatremia [13,14,15]. These side effects are of particular concern in patients with a cardiologic or mixed ATTR phenotype, as well as in those with significant autonomic dysfunction, in whom electrolyte imbalance, orthostatic intolerance, and impaired mobility may further worsen functional status and increase the risk of complications. Therefore, sodium channel-blocking antiepileptic drugs should be prescribed with caution in ATTR patients, with careful assessment of cardiovascular status, autonomic symptoms, and serum sodium levels. Additional second-line therapeutic options for neuropathic pain in ATTR include topical agents, centrally acting analgesics, and weak opioids [9,12]. Topical treatments such as lidocaine and capsaicin may be considered, particularly in patients with localized pain or in those who are unable to tolerate systemic medications [9,12]. These agents offer the advantage of minimal systemic absorption and a favorable safety profile; however, their overall analgesic efficacy is generally modest and often insufficient for moderate-to-severe neuropathic pain. Opioid analgesics, including tramadol and tapentadol, may be employed in selected patients with refractory neuropathic pain [9,12]. These agents combine opioid receptor agonism with additional mechanisms of action, such as inhibition of noradrenaline reuptake, which may enhance their efficacy in neuropathic pain syndromes. Nevertheless, their use should be carefully weighed against the risk of adverse effects, including sedation, constipation, nausea, and potential worsening of autonomic dysfunction, which are particularly relevant in the ATTR population. Paracetamol may also be prescribed, either as monotherapy or in combination with other analgesics; however, its efficacy in neuropathic pain is limited, and it is generally insufficient as a standalone treatment for ATTR-related neuropathic symptoms [1]. As such, paracetamol should be considered primarily as an adjunctive therapy rather than a core component of neuropathic pain management in these patients. Additional therapeutic approaches for neuropathic pain management include agents targeting the N-methyl-D-aspartate (NMDA) receptor, such as ketamine and dextromethorphan [9]. These treatments are generally reserved for highly selected cases, as they often require more invasive routes of administration, including intravenous infusions or intrathecal delivery, and are associated with a non-negligible burden of adverse effects [9]. Other less commonly employed options include cannabinoid-based therapies and intramuscular injections of botulinum toxin type A [9]. Although these interventions may provide benefit in selected patients, their use in ATTR remains limited due to variable efficacy, limited supporting evidence, and concerns regarding tolerability and safety. In addition to pharmacological treatment, a multimodal approach incorporating non-pharmacological interventions may enhance symptom control and improve overall patient outcomes. Physical therapy may help preserve mobility, reduce disability, and mitigate pain-related functional impairment [9]. Psychological interventions, including cognitive behavioral therapy (CBT), acceptance and commitment therapy (ACT), and pain reprocessing therapy, can address the cognitive and emotional dimensions of chronic pain and support adaptive coping strategies [9]. Furthermore, lifestyle interventions—such as reducing pro-inflammatory factors, optimizing sleep hygiene, engaging in regular and tailored physical activity, and limiting alcohol consumption and tobacco use—may contribute to improved pain control and overall quality of life [9]. Integrating these complementary strategies into standard care supports a holistic, patient-centered approach to the management of neuropathic pain in ATTR.

## 4. Orthostatic Hypotension

Autonomic dysfunction is a frequent and highly disabling manifestation of ATTR and may represent the initial clinical presentation in approximately 10% of patients, preceding the onset of sensory–motor neuropathy or cardiomyopathy [16,17]. Among the various features of autonomic involvement, orthostatic hypotension (OH) is one of the most incapacitating and clinically relevant manifestations [16,17]. OH is defined as a sustained decrease in blood pressure upon standing [16,17]. According to standard diagnostic criteria, OH is characterized by a persistent reduction in systolic blood pressure of at least 20 mmHg or in diastolic blood pressure of at least 10 mmHg within 3 min of standing or during head-up tilt to at least 60° [16,17]. In addition, the current European Society of Cardiology (ESC) guidelines on syncope recognize that a systolic blood pressure drop of less than 20 mmHg from the supine to the upright position may also fulfill criteria for OH if the standing systolic blood pressure falls below 90 mmHg [17]. Consensus-based guidelines for the management of OH recommend a stepwise and individualized therapeutic approach [16]. The cornerstone of OH management consists of three sequential steps: identification and correction of aggravating factors, implementation of non-pharmacological interventions, and, when necessary, initiation of pharmacological therapy [16,17]. The first step involves the recognition and correction of reversible or exacerbating factors [16]. Conditions such as anemia should be identified and appropriately treated [16]. In addition, medications that reduce intravascular volume, promote vasodilation, or interfere with sympathetic neurotransmission at the neurovascular junction may worsen OH and related symptoms and should be reviewed critically [16]. These include diuretics, vasodilatory agents such as nitrates and phosphodiesterase-5 inhibitors (e.g., sildenafil), as well as drugs that inhibit norepinephrine release or action, including α-adrenergic blockers, centrally acting α2-agonists, and TCAs [16]. The second step focuses on non-pharmacological measures, which represent the foundation of OH management and should be implemented in all patients [16,17]. Patients should be educated about lifestyle factors that may exacerbate hypotension, including the diuretic effects of caffeine and alcohol, and advised to avoid sugar-rich beverages, such as bottled juices and carbonated soft drinks, due to the hypotensive effects of high-glycemic index carbohydrates [16]. Adequate hydration is essential, with a recommended daily fluid intake of approximately 2.0–2.5 L [16]. Patients should also be encouraged to increase dietary salt intake, for example, by adding 1–2 teaspoons of salt to a balanced diet, provided there are no contraindications [16]. Regular physical activity, tailored to individual tolerance, may improve orthostatic tolerance, while dietary modifications—such as consuming smaller, more frequent meals and reducing carbohydrate content—can help mitigate postprandial hypotension [16]. The third step involves pharmacological treatment, which is generally reserved for patients with persistent or disabling symptoms despite adequate non-pharmacological measures [16,17]. In cases of asymptomatic OH, treatment may not be necessary or may be limited to lifestyle and supportive interventions alone [16,17]. Conversely, when OH is symptomatic and significantly impacts daily functioning, pharmacological therapy is usually required to achieve adequate symptom control and improve quality of life [16,17]. Several pharmacological agents are available for the treatment of OH in patients with ATTR, each with distinct mechanisms of action and safety considerations that should guide individualized therapy. Fludrocortisone (9α-fluorocortisol) is a synthetic mineralocorticoid that increases blood pressure through multiple mechanisms [16]. It promotes renal sodium and water reabsorption, leading to intravascular volume expansion, and enhances vascular responsiveness to endogenous catecholamines and other pressor agents [16]. Despite its efficacy, fludrocortisone should be used with caution in patients with amyloid cardiomyopathy, as it may induce supine hypertension, peripheral oedema, and hypokalaemia [1,16]. In addition, in patients with significant gastrointestinal involvement, fludrocortisone may be poorly tolerated due to potential digestive discomfort, limiting its clinical utility in this subgroup [1,16,17]. Midodrine is an orally administered selective α1-adrenergic receptor agonist that increases blood pressure by inducing peripheral vasoconstriction [16]. Compared with volume-expanding agents, midodrine is generally considered safer in patients with amyloid cardiomyopathy; however, it may still be associated with supine hypertension [1,16]. Furthermore, in patients with pre-existing urinary symptoms or bladder dysfunction, midodrine may not represent the optimal therapeutic choice, as it can increase urinary frequency and exacerbate urinary complaints [1,16]. Droxidopa (l-threo-3,4-dihydroxyphenylserine; l-DOPS) is an oral synthetic amino acid that is converted to norepinephrine by aromatic L-amino acid decarboxylase after systemic absorption [16]. Droxidopa has demonstrated particular efficacy in OH and is generally well tolerated, even in patients with advanced or severe ATTR [16]. Nonetheless, potential adverse effects include supine hypertension, dizziness, nausea, and headache, which warrant careful monitoring during treatment [1,16]. Pyridostigmine, a reversible acetylcholinesterase inhibitor, represents an additional therapeutic option for OH [1,18,19]. By enhancing cholinergic neurotransmission at autonomic ganglia, pyridostigmine amplifies residual sympathetic outflow, resulting in a preferential increase in blood pressure in the upright position, with a lower risk of supine hypertension [18,19]. However, its use may be limited in patients with gastrointestinal involvement, as it is commonly associated with abdominal pain, nausea, vomiting, diarrhea, muscle cramps, and increased salivation [1,18,19]. In addition to pharmacological therapies, mechanical measures such as the use of compression stockings and abdominal binders may provide adjunctive benefit by reducing venous pooling and improving venous return, and should be considered as part of a comprehensive, multimodal management strategy for OH in ATTR [1].

## 5. Diarrhea

Diarrhea represents the most frequently reported gastrointestinal manifestation in patients with ATTR and may significantly impair nutritional status and quality of life [20,21]. First-line pharmacological management typically includes the opioid receptor agonist loperamide, or racecadotril, a peripherally acting enkephalinase inhibitor that reduces intestinal hypersecretion [1,20,21]. While loperamide is widely used and often effective, prolonged treatment may be associated with adverse effects such as nausea and constipation, as well as dizziness and drowsiness, which are particularly undesirable in patients with concomitant OH or autonomic instability [1]. Eluxadoline represents an additional therapeutic option and acts as a mixed μ- and κ-opioid receptor agonist and δ-opioid receptor antagonist, exerting its effects primarily within the enteric nervous system [20,21]. This peripheral mechanism of action may limit central nervous system-related adverse effects, making eluxadoline a potentially suitable option in selected patients with refractory symptoms. In cases where small intestinal bacterial overgrowth (SIBO) is suspected or confirmed, antibiotic therapy may be required [20,21]. Agents such as rifaximin, ciprofloxacin or norfloxacin, and metronidazole or tinidazole can be administered either intermittently or as part of a continuous or rotating regimen, depending on symptom recurrence and clinical response [20,21]. For patients with chronic or severe diarrhea who fail to respond adequately to conventional antidiarrheal therapies, somatostatin analogues—most notably octreotide—may be considered [1,20,21]. These agents reduce intestinal secretion and motility and may provide symptomatic relief in refractory cases [1,20,21]. Nutritional supplementation is essential in patients with evidence of malabsorption or unintentional weight loss and should be initiated as early as possible following the onset of persistent diarrhea to prevent further nutritional deterioration [20,21]. Additional therapeutic measures may include the use of bile acid sequestrants such as cholestyramine, particularly in patients with suspected bile acid malabsorption, in combination with a fat-reduced diet [1,20,21]. Together, these strategies support a comprehensive and individualized approach to the management of gastrointestinal dysautonomia in ATTR.

## 6. Constipation

Constipation is a relatively common gastrointestinal manifestation in patients with ATTR, with a reported prevalence ranging from approximately 20.9% to 40% [20,21]. Similar to chronic diarrhea, constipation significantly contributes to malnutrition and reduced quality of life in this patient population [20,21]. In many cases, severe or obstinate constipation precedes the development of alternating bowel habits, including loose stools and fecal incontinence, and may itself cause substantial discomfort and functional impairment [20,21]. First-line management of constipation in ATTR primarily relies on dietary modifications, including increased fiber intake, and the use of bulking agents and laxatives [1,20,21]. Commonly employed agents include polyethylene glycol, sodium picosulfate, bisacodyl, and lactulose [1,20,21]. In addition, prokinetic agents targeting serotonergic pathways, such as 5-hydroxytryptamine 4 (5-HT4) receptor agonists—including lubiprostone, linaclotide, and prucalopride—may be considered in selected patients [20,21]. Although generally effective, these medications may be associated with adverse effects such as nausea, diarrhea, abdominal cramping, and dyspepsia [20,21]. In some cases, fatigue and dizziness have also been reported, which may exacerbate symptoms of OH when present. Enemas may be used as part of first-line or rescue therapy, particularly in cases of refractory constipation; however, pharmacological interventions require careful dose titration, as excessive treatment may precipitate nausea, abdominal cramping, or diarrhea [20,21]. An additional pharmacological option is pyridostigmine, which may exert prokinetic effects comparable to those of stimulant laxatives such as bisacodyl [22,23]. While pyridostigmine does not appear to improve symptoms in patients with slow-transit constipation, it has demonstrated clinical benefit in individuals with recurrent intestinal pseudo-obstruction, with a relatively favorable safety profile [24]. In this subgroup of patients, pyridostigmine may therefore represent a valuable adjunctive therapeutic option [24].

## 7. Gastroparesis

Early satiety, postprandial fullness, nausea, vomiting, and unintended weight loss are hallmark symptoms of gastroparesis, a condition characterized by delayed gastric emptying in the absence of mechanical gastric outlet obstruction [20,21]. In patients with ATTR, gastroparesis is generally attributed to autonomic dysfunction; however, the precise contribution of efferent sympathetic and parasympathetic pathways remains incompletely understood [20,21]. Neuropathological evidence supports a key role for vagal impairment, as autopsy studies have demonstrated extensive amyloid infiltration and destruction of the vagus nerve and associated ganglia [20,21]. Consistently, functional studies of the oesophagus have revealed motility abnormalities attributable to vagal dysfunction [20,21]. Clinically, gastroparesis has been shown to correlate significantly with reduced modified body mass index (mBMI), and patients with severe gastric retention exhibit markedly lower mBMI values, underscoring the contribution of gastric dysmotility to malnutrition in ATTR [20,21]. First-line pharmacological management typically includes dopamine D2 receptor antagonists, such as domperidone, metoclopramide, and levosulpiride, which enhance gastric motility and exert antiemetic effects [20,21,25,26]. However, their use is limited in ATTR patients by important safety concerns. These agents are contraindicated in the presence of QTc prolongation, concomitant use of QT-prolonging medications, or pre-existing cardiac disease, including heart failure, making them poorly suited for patients with amyloid cardiomyopathy [20,21,25,26]. Moreover, D2 antagonists may induce extrapyramidal symptoms and parkinsonism, potentially worsening gait disturbances in patients with ATTR-PN [20,21,25,26]. Additional adverse effects, including drowsiness, fatigue, and dizziness, further limit their tolerability in this population [20,21]. Erythromycin represents an alternative therapeutic option due to its prokinetic properties mediated by motilin receptor stimulation, which can enhance gastric emptying [20,21,27,28]. In addition, erythromycin may exert appetite-stimulating effects, possibly through central mechanisms, thereby improving early satiety in patients with prominent gastrointestinal involvement [20,21,27,28]. Nevertheless, its use should be limited to short-term or intermittent courses, as chronic administration is associated with tachyphylaxis and an increased risk of adverse effects. Other pharmacological options explored in gastroparesis include 5-hydroxytryptamine 4 (5-HT4) receptor agonists, such as prucalopride, and ghrelin receptor agonists, including relamorelin, which may improve gastric motility and symptom burden in selected patients [20,21]. For the management of prominent nausea and vomiting, ondansetron may be considered; however, its potential to prolong the QTc interval renders it unsuitable for patients with amyloid cardiomyopathy, and concomitant administration with apomorphine should be avoided due to the risk of severe hypotension [1,20,21]. To address appetite loss and weight decline, mirtazapine—a noradrenergic and specific serotonergic antidepressant—may be employed as an adjunctive therapy [1]. In addition to its appetite-stimulating properties, mirtazapine may exert antiemetic effects [1]. Nonetheless, clinicians should be aware that mirtazapine can exacerbate orthostatic hypotension and may cause xerostomia, nausea, and constipation, necessitating careful patient selection and monitoring [1].

## 8. Sexual Dysfunction

Sexual dysfunction is highly prevalent in patients with ATTR, affecting up to approximately 40% of male patients and up to 42% of female patients, and may occur early in the course of the disease [29,30]. The pathogenesis of sexual dysfunction in ATTR is multifactorial, involving a complex interplay of neurogenic, vasculogenic, myogenic, and psychological mechanisms, often in the context of generalized autonomic dysfunction and multisystem involvement [29,30]. In male patients, sexual dysfunction may manifest as erectile dysfunction, retrograde ejaculation, or azoospermia [29]. Phosphodiesterase inhibitors have been reported to be effective and generally safe for the treatment of erectile dysfunction in ATTR [1,29]. However, phosphodiesterase type 5 inhibitors (PDE5i) should be prescribed with caution, particularly in patients with documented OH, as these agents may further reduce blood pressure and exacerbate autonomic symptoms [1,29]. In cases of inadequate response or intolerance to PDE5i, alternative therapeutic options include vacuum erection devices, intraurethral alprostadil, intracavernous injections, and penile prosthesis implantation [1,29]. For the management of premature ejaculation, dapoxetine remains the only pharmacological treatment approved by regulatory authorities [29]. Other therapeutic options, such as selective serotonin reuptake inhibitors, topical anesthetic agents, and tramadol, may be considered off-label in selected patients [29]. Across male sexual dysfunctions, the combination of pharmacological treatment with psychological and behavioral interventions may enhance therapeutic outcomes and improve patient satisfaction [29]. In female patients, sexual dysfunction encompasses a broad spectrum of disorders, including sexual arousal disorder, hypoactive sexual desire disorder, impaired vaginal lubrication, orgasmic dysfunction, and dyspareunia [29]. Cognitive behavioral therapy represents the cornerstone of management for female sexual dysfunction and should be integrated into routine care whenever feasible [29]. Flibanserin, a non-hormonal centrally acting agent, has recently emerged as the first approved pharmacological treatment for women with hypoactive sexual desire disorder [29,31]. However, its use in patients with ATTR warrants careful consideration. Flibanserin has been associated with adverse effects such as hypotension and dizziness, which may exacerbate symptoms in patients with concomitant OH [31]. In addition, gastrointestinal side effects, including nausea and abdominal discomfort, have been reported, potentially limiting its tolerability in patients with ATTR-related gastrointestinal involvement [31]. Consequently, flibanserin should be prescribed with caution in this population, with careful assessment of autonomic and gastrointestinal symptoms prior to treatment initiation. In contrast, testosterone therapy remains the only intervention supported by level 1 evidence for the treatment of sexual arousal disorder in women [29]. For female patients with orgasmic disorder, directed masturbation is considered the preferred therapeutic strategy [29].

## 9. Urinary Dysfunction

The prevalence of lower urinary tract symptoms (LUTS) in patients with ATTR is high, affecting up to 83% of this population [29,30,32]. Emerging evidence suggests that lower urinary tract dysfunction (LUTD) may occur at early stages of the disease [29,30,32]. Voiding symptoms are the most frequently reported LUTS, with 34.8–87.5% of patients experiencing at least one voiding symptom, such as hesitancy, straining, and/or intermittency [29]. Underactive bladder is defined as a symptom complex suggestive of detrusor underactivity and is typically characterized by prolonged voiding time, with or without a sensation of incomplete bladder emptying, often accompanied by hesitancy, reduced bladder sensation, and a weak urinary stream [29]. Incomplete bladder emptying predisposes patients to urinary tract infections (UTIs), which have been reported in up to 50% of individuals with ATTR [29]. Due to impaired bladder sensation, bacteriuria may remain asymptomatic in a significant proportion of patients [29]. Elevated post-void residual (PVR) volumes also increase the risk of upper urinary tract damage, and cases of hydronephrosis in ATTR patients have been described [29,32]. Urinary incontinence is another common manifestation, reported in 16.7–37.5% of patients [29]. Other urinary symptoms, such as increased or decreased urinary frequency and urgency, have also been described but appear to be less common and likely less disease-specific than underactive bladder and stress urinary incontinence [29]. In early disease stages, when bladder sensation is reduced but detrusor contractility is preserved, behavioral strategies such as timed voiding or Valsalva voiding may be beneficial [29]. Patients should be carefully counseled regarding the potential risk of pelvic organ prolapse associated with Valsalva voiding, particularly in neurogenic populations [29]. Clean intermittent catheterization (CIC) remains the standard of care for patients with detrusor underactivity and chronic urinary retention [29]. In individuals with symptomatic elevated PVR volumes exceeding 300–400 mL, CIC should be initiated [29]. A suprapubic catheter may represent an alternative in patients with significant upper limb motor impairment and should be preferred over an indwelling urethral catheter due to its lower risk of UTIs and urethral complications [29]. In selected patients with non-obstructive urinary retention, sacral neuromodulation may be considered as an alternative therapeutic option; notably, there is no contraindication to sacral nerve stimulation in the presence of dysautonomia [29]. In rare cases where CIC is not feasible and life expectancy is long, advanced reconstructive procedures—such as continent catheterizable channels or ileal conduits—may be considered in specialized tertiary referral centers [29]. In cases of stress urinary incontinence, duloxetine, an SNRI, may be used [29]. Duloxetine acts at Onuf’s nucleus by increasing the activity of pudendal motor neurons, thereby enhancing urethral sphincter tone and promoting detrusor relaxation [29]. When conservative measures fail, surgical intervention may be required [29]. Surgical options for stress urinary incontinence in men include bulking agents, slings, adjustable periurethral balloons, and artificial urinary sphincter implantation [29,30]. In women, available procedures include mid-urethral slings, bulking agents, fascial slings, adjustable periurethral balloons, and, in selected countries, artificial urinary sphincter devices [29,30]. Poor bladder compliance is associated with a high risk of upper urinary tract deterioration [29,30]. Antimuscarinic agents may be used as first-line therapy, while intradetrusor botulinum toxin injections represent a second-line option in cases of treatment failure [29,30]. There is no absolute contraindication to antimuscarinic therapy in patients with dysautonomia; however, the severity of cardiac and gastrointestinal involvement should be carefully assessed within a multidisciplinary framework prior to treatment initiation [29,30]. Desmopressin, a synthetic analogue of arginine vasopressin with antidiuretic properties, is generally considered the treatment of choice for nocturia [29,33]. Several clinical trials have demonstrated its efficacy in reducing nocturnal urine production and the number of nocturnal voids [29,33]. Serum sodium levels should be closely monitored during desmopressin therapy, as severe hyponatremia may occur [29,33].

## 10. Ophthalmological Manifestations

The prevalence and spectrum of ocular manifestations in ATTRv vary according to the specific TTR mutation and geographic distribution. Early reports estimated ophthalmological involvement in approximately 8% of cases; however, more recent studies describe ocular manifestations in 10–24.1% of patients with ATTRv [34,35,36,37,38]. The most frequently reported abnormalities include reduced tear break-up time and pathological Schirmer test results, observed in 79.5% and 67% of patients, respectively [34,35,36,37,38]. Other common findings include amyloid deposition in the iris (38.4%) and in the anterior lens capsule (32.9%), scalloped iris configuration (27.9%), glaucoma (20%), vitreous amyloidosis (17.4%), abnormal conjunctival vessels (14%), and retinal amyloid angiopathy (4%) [34,35,36,37,38]. Ocular involvement in ATTRv can be classified according to three main pathophysiological mechanisms: direct and indirect consequences of sensory and autonomic neuropathy; direct and secondary effects of systemic and local ocular transthyretin deposition; and vascular ocular lesions related to altered TTR production [34,35,36,37,38]. Management of ocular manifestations in ATTR is largely condition-specific and follows standard ophthalmological practice [1]. Surgical interventions are frequently required, particularly for complications such as vitreous amyloidosis or glaucoma [1]. In patients with dry eye disease, symptomatic treatment with preservative-free artificial tears is recommended, along with adjunctive measures such as nighttime eye ointments or gels and the use of nighttime moisture masks to reduce corneal exposure and improve ocular surface protection [1].

## 11. Limitations

Despite the growing body of literature on ATTR, relatively few studies specifically address the symptomatic management of this condition. Most available research has primarily focused on disease-modifying therapies aimed at slowing or halting disease progression, such as TTR stabilizers and gene-silencing agents, rather than on the treatment of the multisystem symptoms that significantly affect patients’ quality of life. Moreover, there are currently no dedicated, evidence-based clinical guidelines specifically addressing the symptomatic treatment of ATTR. As a result, therapeutic decisions in daily clinical practice are often extrapolated from studies conducted in other neurological, gastrointestinal, urological, or cardiological disorders, or are based on expert opinion rather than on high-quality randomized controlled trials. Consequently, a substantial proportion of the information presented in this review is derived from observational studies, small case series, expert consensus, and interdisciplinary clinical experience, including personal observations from the management of patients with ATTR in real-world settings. It should be noted that, for many symptomatic interventions discussed in this review, high-quality randomized trials in ATTR patients are lacking. Available evidence largely comes from expert consensus, retrospective observational studies, or case series. Despite the limited strength of evidence, these interventions have been reported to provide clinical benefit in many patients. Nevertheless, larger controlled studies are needed to more rigorously assess efficacy and safety, and to inform stronger, evidence-based recommendations. This heterogeneity in data sources may limit the generalizability of some recommendations and highlights the lack of standardized treatment algorithms. Finally, the multisystemic and heterogeneous nature of ATTR, along with variability related to genotype, disease stage, and organ involvement, further complicates the design of large, controlled studies focused on symptomatic treatment. These limitations underscore the urgent need for prospective studies and consensus-driven guidelines to better define optimal symptomatic management strategies and improve patient-centered outcomes in ATTR.

## 12. Conclusions and Future Perspectives

ATTR is a complex, multisystem disorder in which neurological, cardiac, gastrointestinal, urogenital, sexual, and ophthalmological manifestations significantly contribute to disease burden and impairment of quality of life. While major advances have been achieved in disease-modifying therapies, symptomatic manifestations often persist and may even progress despite etiological treatment, highlighting the ongoing need for comprehensive symptomatic management. This review underscores the importance of early recognition and proactive treatment of ATTR-related symptoms, which frequently arise early in the disease course and may precede overt organ dysfunction. Symptomatic therapies (Table 1), although largely supported by limited evidence, remain essential to preserve nutritional status, functional independence, and overall well-being. A multidisciplinary and individualized approach is therefore crucial, taking into account autonomic dysfunction, comorbidities, and the frequent coexistence of cardiac and neurological involvement. Future research should increasingly focus on the development of structured, evidence-based symptomatic treatment algorithms tailored to ATTR. Prospective studies and randomized controlled trials specifically addressing symptomatic interventions are urgently needed, particularly in the context of autonomic dysfunction, gastrointestinal complications, urogenital disorders, and pain management. Furthermore, the integration of patient-reported outcomes and quality-of-life measures into clinical trials may help better capture the real-world impact of symptomatic treatments. Finally, as survival improves with disease-modifying therapies, long-term management of symptoms will become an even more critical component of care. Establishing consensus guidelines, fostering interdisciplinary collaboration, and promoting ATTR-specific symptomatic research will be essential steps toward optimizing holistic, patient-centered care in this evolving therapeutic landscape.

## Figures and Tables

**Table 1:** Symptomatic management of transthyretin amyloidosis (ATTR): therapeutic options and clinical caveats. 5-HT: 5-hydroxytryptamine. ATTR: transthyretin amyloidosis. NMDA: N-methyl-D-aspartate. PDE5i: Phosphodiesterase type 5 inhibitors. SNRIs: serotonin–norepinephrine reuptake inhibitors.

Symptom	Available Treatments	Clinical Notes/Precautions
Pain	Gabapentinoids (Pregabalin, Gabapentin) Tricyclic Antidepressants (Amitriptyline, Nortriptyline) Serotonin–noradrenaline reuptake inhibitors (SNRIs) (Venlafaxine, Duloxetine) Sodium-channel blockers (Carbamazepine, Oxcarbazepine) Topical treatments (Lidocaine, Capsaicin) Opioid analgesics (Tramadol, Tapentadol) Paracetamol Agents targeting the N-methyl-D-aspartate (NMDA) receptor (Ketamine, Dextromethorphan) Cannabinoid-based therapies Intramuscular injections of botulinum toxin type A Psychological interventions (Cognitive behavioral therapy, acceptance and commitment therapy, pain reprocessing therapy) Lifestyle interventions (Reducing pro-inflammatory factors, optimizing sleep hygiene, engaging in regular and tailored physical activity, and limiting alcohol consumption and tobacco use)	Gabapentinoids: caution in patients with orthostatic hypotension and ambulatory issues Tricyclic Antidepressants: caution in patients with a predominantly cardiologic or mixed ATTR phenotype SNRIs: caution in patients with a predominantly cardiologic or mixed ATTR phenotype Sodium-channel blockers: caution in patients with a predominantly cardiologic or mixed ATTR phenotype, and in patients with orthostatic hypotension Topical treatment: insufficient for moderate-to-severe neuropathic pain Opioid analgesics: caution in patients with orthostatic hypotension and gastrointestinal issues Paracetamol: limited efficacy in neuropathic pain Agents targeting the NMDA receptor: invasive route of administration Cannabinoid-based therapies and intramuscular injections of botulinum toxin type A: variable efficacy
Orthostatic Hypotension	Non-pharmacological interventions: ○ Recognition and correction of reversible or exacerbating factors ○ Considering withdrawing medications that reduce intravascular volume, promote vasodilation, or interfere with sympathetic neurotransmission at the neurovascular junction ○ Correcting lifestyle factors that may exacerbate hypotension ○ Adequate hydration ○ Increasing dietary salt intake ○ Regular physical activity Pharmacological interventions: ○ Fludrocortisone ○ Midodrine ○ Droxidopa ○ Pyridostigmine Mechanical measures (Compression stockings, abdominal binders)	Fludrocortisone: caution in patient with amyloid cardiopathy and gastrointestinal issues Midodrine: caution in patients with urinary dysfunctions Droxidopa: caution in patients with autonomic dysfunction Pyridostigmine: caution in patients with gastrointestinal issues
Diarrhea	Loperamide Racecadotril Eluxadoline Antibiotic therapy (Rifaximin, Ciprofloxacin, Norfloxacin, Metronidazole, Tinidazole) Somatostatin analogues (Octreotide) Nutritional supplementation Bile acid sequestrants (Cholestyramine)	Loperamide: caution in patients with orthostatic hypotension Eluxadoline: suitable option for selected patients with refractory symptoms Antibiotic therapy: in cases of small intestinal bacterial overgrowth Somatostatin analogues: in case of severe and chronic diarrhea
Constipation	Dietary modifications (Increased fiber intake) Bulking agents and laxatives (Polyethylene glycol, Sodium picosulfate, Bisacodyl, Lactulose) 5-hydroxytryptamine 4 (5-HT4) receptor agonists (Prucalopride, Lubiprostone, Linaclotide) Enemas Pyridostigmine	5-HT4 receptor agonists: caution in patients with orthostatic hypotension Enemas: in cases of refractory constipation
Gastroparesis	Dopamine D2 receptor antagonists (Domperidone, Metoclopramide, Levosulpiride) Erythromycin 5-HT4 receptor agonists (Prucalopride, Lubiprostone, Linaclotide) Ghrelin receptor agonists, (Relamorelin) Ondansetron Mirtazapine	Dopamine D2 receptor antagonists: caution in patients with amyloid cardiopathy, gait issues and orthostatic hypotension Erythromycin: appetite-stimulant and prokinetic drug 5-HT4 receptor agonists: caution in patients with orthostatic hypotension Ondansetron: treatment of nausea and vomiting; caution in patient with amyloid cardiopathy Mirtazapine: appetite-stimulant and antiemetic drug; caution in patients with orthostatic hypotension
Sexual Dysfunction	Male patients: ○ Phosphodiesterase type 5 inhibitors (PDE5i) (Sildenafil) ○ Vacuum erection devices ○ Intraurethral alprostadil ○ Intracavernous injections ○ Penile prosthesis implantation ○ Dapoxetine ○ Selective serotonin reuptake inhibitors ○ Topical anesthetic agents ○ Tramadol Female patients: ○ Cognitive behavioral therapy ○ Flibanserin ○ Testosterone therapy ○ Direct masturbation	PDE5i: caution in patients with orthostatic hypotension Selective serotonin reuptake inhibitors, Topical anesthetic agents, Tramadol: off-label treatments Flibanserin: caution in patients with autonomic dysfunction and gastrointestinal issues
Urinary Dysfunction	Behavioral strategies (Timed voiding, Valsalva voiding) Clean intermittent catheterization Suprapubic catheter Sacral neuromodulation Advanced reconstructive procedures (Continent catheterizable channels or ileal conduits) Duloxetine Surgical options for stress urinary incontinence (Bulking agents, slings, adjustable periurethral balloons, artificial urinary sphincter implantation, in male patients; mid-urethral slings, bulking agents, fascial slings, adjustable periurethral balloons, artificial urinary sphincter devices, in female patients) Antimuscarinic agents Intradetrusor botulinum toxin Desmopressin	Valsalva voiding: risk of pelvic organ prolapse Antimuscarinic agents: caution in patients with amyloid cardiopathy and gastrointestinal issues Desmopressin: treatment of nocturia; risk of hyponatremia
Ophthalmological Manifestations	Surgical interventions Dry eye: preservative-free artificial tears, nighttime eye ointments or gels, nighttime moisture masks	Management of ocular manifestations in ATTR is largely condition-specific and follows standard ophthalmological practice

## Data Availability

No new data were created or analyzed in this study.
